# Conservative Management of a Case of Peripartum Cardiomyopathy in a Young Multigravida

**DOI:** 10.7759/cureus.55928

**Published:** 2024-03-11

**Authors:** Priya R Nair, Snehal S Deshmukh, Preeti R Gattani, Anupama V Dhobale

**Affiliations:** 1 Obstetrics and Gynaecology, Datta Meghe Medical College, Datta Meghe Institute of Higher Education and Research (DU), Nagpur, IND

**Keywords:** pulmonary edema, peripartum cardiomyopathy, immediate postpartum period, dilated cardiomyopathy, maternal morbidity, maternal mortality

## Abstract

Peripartum cardiomyopathy (PPCM) is a rare disorder that generally affects the elderly multigravida females. It is a type of dilated cardiomyopathy that generally affects the last trimester of pregnancy or early postpartum period. Several risk factors are associated with the development of PPCM. Even though PPCM has greater morbidity, if managed promptly, it can be reverted with minimal morbidity or mortality. We present a case of a young woman, multigravida, with moderate anemia corrected, who was taken for emergency lower segment cesarean section, without previous cardiac evaluation, and ended up with pulmonary edema intraoperatively. Later on, her evaluation was done which came out to be PPCM. She was managed conservatively thereafter with no significant morbidity and a good maternal and perinatal outcome. We should be alert in diagnosing a case of PPCM with prompt treatment to reduce mortality. Cardiovascular conditions cause approximately 26 percent of pregnancy-related deaths which include valvular heart disease and congenital heart disease. Appropriate diagnosis and management are necessary for preventing mishaps.

## Introduction

Peripartum cardiomyopathy (PPCM) is a new onset systolic dysfunction that manifests during the peripartum period, affecting approximately one in every three thousand to four thousand live births and impacting thousands of women in developing countries annually [1].

The diagnostic criteria for PPCM encompass several key indicators. Firstly, the manifestation of cardiac failure symptoms must occur during the last trimester of pregnancy or within five months of delivery. Additionally, there must be an absence of any identifiable cause for the diagnosed cardiac failure. Another significant criterion involves the lack of recognizable heart disease before the last month of pregnancy. Furthermore, echocardiographic findings play a pivotal role in confirming PPCM diagnosis, requiring specific parameters to be met. These echocardiographic criteria include a left ventricular end-diastolic dimension of ≥2.7 centimeter/meter square (cm/m^2^), motion mode (M-mode) fractional shortening of ≤30 percent (%), and a left ventricular ejection fraction of ≤0.45. These comprehensive criteria serve as essential guidelines for healthcare professionals in accurately identifying and diagnosing PPCM [2,3].

The features of PPCM often imitate those of other peripartum conditions such as pulmonary embolism or eclampsia, potentially leading to delay in diagnosis and management. It is crucial to maintain vigilance in accurately diagnosing cases of cardiomyopathy. A collaborative effort involving an anesthetist, obstetrician, intensivist, and physiotherapist is imperative to save the life of a patient facing an acute crisis. In this context, we present a unique case of a young multigravida who remained asymptomatic throughout her pregnancy and had moderate anemia that was successfully corrected through blood transfusion. However, this correction acted as a trigger for her weak heart, subsequently leading to the development of labor pains and necessitating emergency cesarean section, during which she developed pulmonary edema. Later on, the investigations confirmed the diagnosis of PPCM and she was successfully managed postoperatively by the expert team.

## Case presentation

A 26-year-old, third gravida, with the first being a full-term normal vaginal delivery and the second a lower segment cesarean section, presented to our outpatient department at 37 weeks. The patient was unbooked and referred from a peripheral center for correction of moderate anemia with hemoglobin (Hb) 7 grams/deciliter (gm/dl) and exhibited no major symptoms. Her medical, surgical, and family history were unremarkable.

On general examination, the patient was thin-built, afebrile, showed no signs of malnutrition but presented with significant pallor. There was no edema, lymphadenopathy, icterus, cyanosis, or clubbing. Although there were no overt signs of failure, a soft systolic murmur was noted in the mitral and tricuspid areas. Tachycardia was present with a pulse rate of 110 per minute, with a blood pressure of 110/74 millimeters of mercury (mm Hg). Chest examination revealed clear lung fields, and the uterus, corresponding to 34 weeks of gestation, exhibited mild intrauterine growth restriction. Fetal heart sounds were normal, and the previous cesarean scar appeared normal.

The patient was asymptomatic with no restriction in day-to-day activities and admitted that she was non-compliant with iron and calcium tablets during the antenatal period. Following admission, complete blood count, peripheral smear (PS), and iron profile were performed, revealing a hemoglobin level of 6.9 gm/dl with microcytic hypochromic red cells on PS. She received one unit of packed red blood cells and was scheduled for a two-dimensional (2D) echo and Doppler ultrasound of the fetus the next day.

However, due to monetary constraints, the patient left against medical advice the following day without undergoing further evaluation. A week later, she presented to the emergency department in labor, reporting swelling and breathlessness after the previous blood transfusion. Examination revealed elevated blood pressure of 140/86 mm Hg, blood oxygen saturation (SpO_2_) of 95 percent (%), a murmur, clear lungs, edema in the feet, and increased fetal heart sounds. In labor, with a cervical dilation of 3 centimeters (cm) and 60% effacement, she underwent an emergency lower segment cesarean section (LSCS) under general anesthesia. She delivered a male child of 2.4 kilograms (Kg) baby weight. The baby was evaluated by a pediatrician and handed over to the relatives.

**Figure 1:**
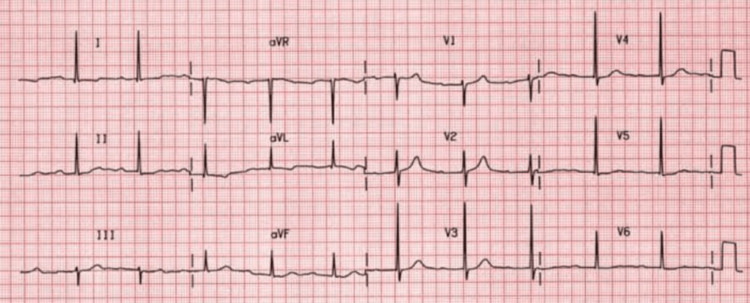
ECG showing first-degree atrioventricular (AV) block

Postdelivery, the patient experienced a sudden drop in SpO_2_ to 70%, leading to pulmonary edema. Intraoperative administration of injection (inj.) furosemide and subsequent management in the intensive care unit (ICU) on bilevel positive airway pressure (BiPap) were initiated. Electrocardiography (ECG) revealed a first-degree atrioventricular block as shown in Figure 1. 

**Figure 2:**
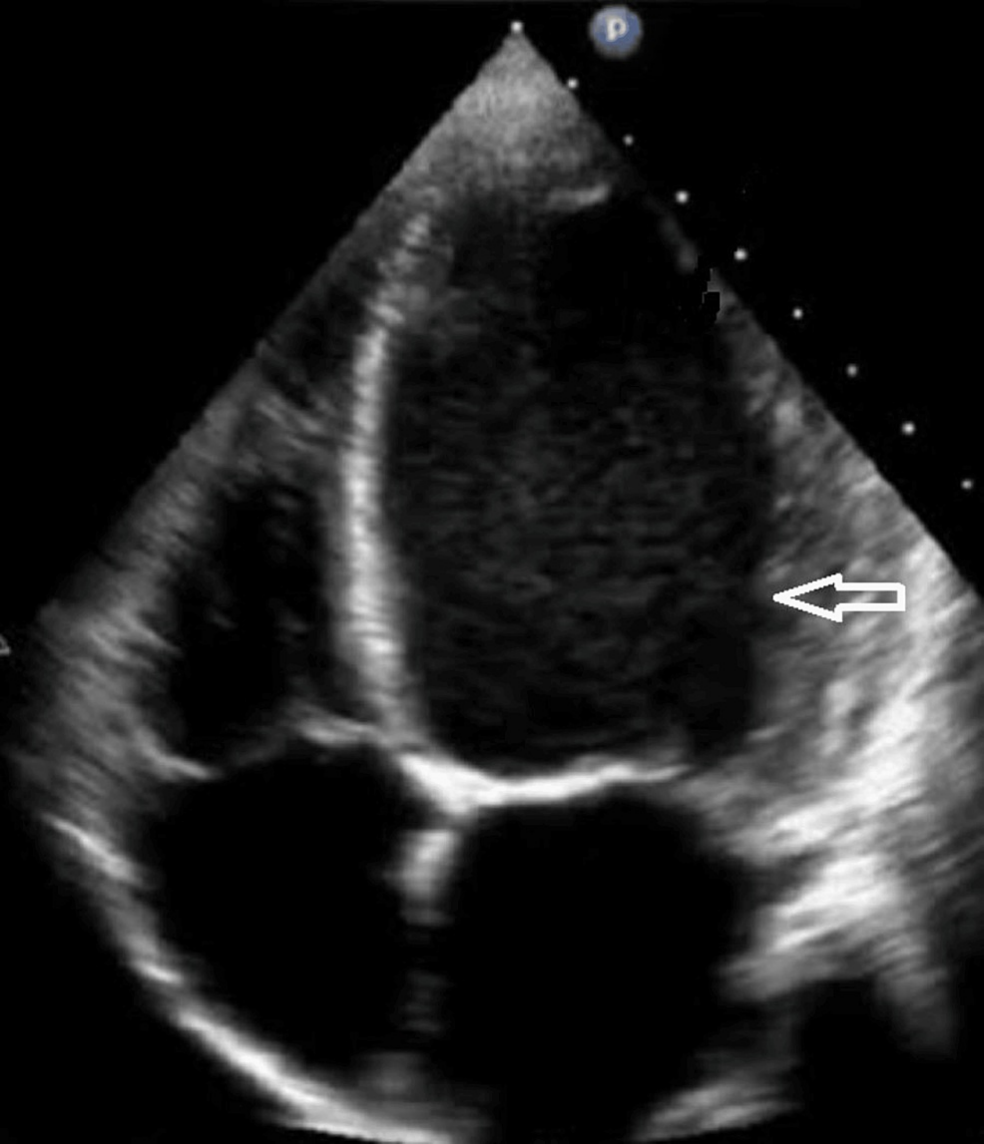
2D echo of the patient The arrow shows the dilated left ventricle 2D: Two-dimensional

2D echo indicated a dilated left ventricle with hypokinesia, jerky septal motion, and an ejection fraction of 30%, along with congested inferior vena cava as shown in Figure 2. A diagnosis of peripartum cardiomyopathy was made, and conservative management with oral furosemide and amlodipine for elevated blood pressure was implemented.

The patient was discharged uneventfully on day 10 post-LSCS with a healthy baby. A follow-up with a cardiologist at four weeks showed an improved ejection fraction of 45%, and the patient was advised on hemoglobin build-up and medication continuation.

## Discussion

PPCM represents a rare form of delayed myopathy characterized by an unknown origin. Unlike hereditary or genetic forms of cardiomyopathy, PPCM is non-hereditary and non-genetic. Several identified risk factors include multiparity, black race, advanced maternal age, smoking, alcohol consumption, malnutrition, and pre-eclampsia [1,4]. The autoimmune response of fetal cells in maternal circulation and cardiac tissue is also hypothesized as a potential cause of PPCM [3]. It often manifests with symptoms such as dyspnea, fatigue, and edema, which may mimic normal pregnancy and peripartum states as well as other pregnancy-related co-morbidities such as pulmonary emboli, upper respiratory tract infections, or severe pre-eclampsia. This mimicry can lead to a delay in diagnosis and under-recognition of the condition [5]. In our case, a notable limitation was the absence of a 2D echo evaluation, as the patient underwent emergency LSCS without prior cardiac assessment.

Various etiologies for PPCM have been proposed, including the cardiovascular stress of pregnancy due to fluid overload or myocarditis [6]. Some researchers have explored the correlation between PPCM and an inflammatory response during pregnancy, indicated by elevated levels of tumor necrosis factor-alpha and interleukin-6 [7,8]. Selenium deficiency has also been suggested as a potential cause for PPCM [9,10]. While specific laboratory abnormalities for PPCM are not well-defined, brain natriuretic peptide levels are often elevated. Other diagnostic tests include cardiac enzyme assessment, as well as imaging studies such as ECG, chest X-ray, and 2D echo. ECG may reveal sinus tachycardia, nonspecific ST and T wave abnormalities, and voltage abnormalities [11]. Chest X-rays may show signs of pulmonary edema, congestion, broncho-vascular markings, pleural effusion, cardiac enlargement, and obliteration of costophrenic angles [12]. Echocardiography typically indicates decreased left ventricular contractility, left ventricular hypokinesia, and enlargement without hypertrophy [13].

Treatment for PPCM involves fluid and salt restriction, beta blockers, diuretics, and digoxin. Hydralazine can be considered during pregnancy to reduce afterload. Diuretics should be used cautiously to prevent dehydration and placental insufficiency. Angiotensin-converting enzyme inhibitors and angiotensin receptor blockers are contraindicated during pregnancy. Exercise is encouraged based on the patient's heart condition, and early use of anticoagulants may be considered to prevent coagulation issues [14]. A second pregnancy is generally not recommended due to a 30% increased risk of PPCM in subsequent pregnancies, posing greater risks to both the patient and the child. While heart transplant is an option for those unresponsive to medical management, the current rate of heart transplants varies between 4% and 7% due to improved medical interventions [15].

## Conclusions

Pregnant women may present with PPCM in a varied manner. Several cases of PPCM are quoted in the literature. Some had co-morbidity like gestational hypertension, some with no morbidity but with incidental findings on 2D echo suggestive of PPCM. Our case was unique as the patient was symptomatic throughout pregnancy and anemia correction along with stress of LSCS was the triggering factor for the development of pulmonary edema later leading to the diagnosis of PPCM. It is a rare but serious condition affecting women of childbearing age group. Correct diagnosis and prompt treatment with a multidisciplinary approach can help save the life of the mother. Despite such high morbidity and greater chances of recurrence in subsequent pregnancy, it recovers within three to six months of onset. Early diagnosis of a high-risk pregnancy with proper evaluation will help detect the case. The patient should always be managed in a tertiary care center as they have a high chance of landing in an emergency, to prevent maternal morbidity and mortality.
